# Measurable residual disease is not a universally reliable surrogate for progression-free survival in clinical trials of new chronic lymphocytic leukemia therapies

**DOI:** 10.1038/s41375-026-03012-2

**Published:** 2026-07-07

**Authors:** Florian Simon, Othman Al-Sawaf

**Affiliations:** Faculty of Medicine and University Hospital Cologne, Department I of Internal Medicine, Center for Integrated Oncology Aachen Bonn Cologne Duesseldorf, German CLL Study Group, University of Cologne, Cologne, Germany

**Keywords:** B-cell lymphoma, Chronic lymphocytic leukaemia

## To the Editor:

We read with interest the recent article by Wang and colleagues, titled “Measurable residual disease is not a universally reliable surrogate for progression-free survival in clinical trials of new chronic lymphocytic leukemia therapies,” published in Leukemia [1]. Drawing on a meta-analysis of 43 trials and 9628 subjects, the authors report that, although detectable measurable residual disease (MRD) is associated with a higher individual-level risk of progression or death (HR 3.67, 95% CrI 3.34–4.03), trial-level surrogacy of MRD for progression-free survival (PFS) is weak overall (Spearman *R* = −0.33; *R*² = 0.06). They conclude that the validity of MRD as a surrogate for PFS is context-dependent and should be interpreted cautiously when informing clinical or regulatory decisions. We commend the authors for tackling a question of considerable contemporary importance to CLL trial design and drug regulation. Nevertheless, several critical questions remain regarding the methodology and interpretation of the analyses, which we believe merit further consideration.

A first concern relates to the composition and transparency of the dataset on which the analyses are based. The headline figure of 43 trials in the abstract is not reconciled with the later described datapoints from both the text or figures: the methods describe an individual-level analysis of 4636 subjects from 28 trials and a trial-level analysis of 29 therapy comparisons from 18 trials, while the CONSORT-style flow diagram (Figure 1) reports “43 Analyzed” decomposed into “32 for individual level analysis” and “18 for trial level analysis.” The supplementary trial list contains more than 40 entries, with the supplement noting that some articles originate from the same clinical trial but were included separately because they reported different types of data; conversely, at least one trial that clearly contributes a data point to Figure 5—the MRD-guided ibrutinib–venetoclax arm of FLAIR [2] - does not appear in the supplementary trial list. We would therefore appreciate clarification of the actually included number of trials/comparison as well as patient numbers for each analysis and how overlap between trials might have been adjusted in the independence-adjusted weighting.

A second concern lies with the selection and pooling of studies. The pooled individual-level HR of 3.67 is derived from trials encompassing first-line trials within a fixed-duration setting such as CLL14 [3] and GLOW [4] as well as relapsed/refractory disease such as MURANO [5], alongside single-arm phase 2 trials without comparator arms. These trials differ substantially with regard to their included populations, therapy setting and study phase and in some cases included experimental or even ineffective regimens, such as mitoxantrone, which does not reflect an established regimen in CLL at any time. While the authors describe an independence-adjusted weighting scheme to address comparisons arising from multi-arm trials, several trials nonetheless contribute multiple comparisons to the trial-level analysis (CLL13: 7 of 29; AMPLIFY, ELEVATE-TN, and FLAG CLL2: 3 each). Moreover, the association between end-of-treatment MRD status and PFS is strongly dependent on observation time: virtually all randomized trials with adequate post-treatment follow-up after contemporary fixed-duration treatments have demonstrated an MRD-PFS correlation [3, 4, 6, 7]. Notable exceptions include the AMPLIFY [8] study, which has limited post-treatment follow-up and carries competing survival risk from Covid-19-related PFS events, and the CLL17 study, in which landmark MRD analyses were not reported due to limited post-treatment follow-up at the time of the primary analysis [9].

A further specific concern in this regard is the inclusion of the MRD-guided ibrutinib–venetoclax arm of FLAIR [2] in the trial-level analysis (FLAIR: IVe v FCR in Figure 5). By trial design, treatment duration in this arm was determined by MRD status itself. Including such a comparison in an analysis intended to validate MRD as a surrogate for PFS appears problematic to us. We would welcome the authors’ rationale for retaining this comparison, and an indication of whether the headline correlation is materially altered by its exclusion.

A third concern is the heterogeneity of MRD assessments pooled in the primary trial-level analysis. The headline correlation of *R* = −0.33 is derived from 29 comparisons in which the definition and timing of MRD assessment differ inherently across the included trials: some report landmark PFS results from end-of-treatment MRD, such as CLL14 [3] and CLL13 [6], while others report on-treatment MRD during continuous therapy. End-of-treatment MRD after fixed-duration venetoclax-based therapy and on-treatment MRD during continuous BTK-inhibitor therapy reflect different points in fundamentally different disease trajectories, and we are uncertain that pooling them into a single primary analysis is appropriate. The supplementary sensitivity analyses do stratify by MRD assessment at 9 and 15 months from start of therapy, but these remain inherently different between treatment paradigms and they do not inform the primary trial-level estimate on which the authors’ main conclusion rests. We wonder whether restricting the primary analysis to a treatment-paradigm-appropriate, harmonized MRD timepoint definition would more precisely inform on the validity of the surrogate endpoint.

Finally, the authors cite the IQWiG/Buyse [10, 11] framework for establishing the validity of a surrogate endpoint, which requires individual-level surrogacy to be established before trial-level surrogacy can be meaningfully assessed. However, individual-level surrogacy has not been established for continuous BTK-inhibitor monotherapy—indeed, the long-term follow-up of E1912 demonstrated that detectable MRD does not preclude prolonged PFS in ibrutinib-treated patients [12], and this trial is itself among those included in the present dataset. Comparisons involving continuous BTKi therapy (E1912, FLAIR IR vs FCR, HELIOS, GENUINE, ELEVATE-TN) account for a substantial proportion of the trial-level data points despite this unresolved prerequisite. Thus, reporting a weak correlation in a setting where the individual-level link itself has not been established risks misattributing the claim of missing surrogacy.

In conclusion, we applaud the authors’ effort in providing further data on the validity of MRD as a surrogate endpoint for PFS in clinical trials of CLL. Previous analyses on surrogacy with more stringent methodology have confirmed strong association between MRD and PFS on a patient- as well as trial level for fixed-duration therapies [13], while MRD status does not correlate with outcomes in patients on continous BTKi. Nevertheless, we believe the questions outlined above—regarding the transparency of the included dataset, the heterogeneity of pooled studies and MRD timepoints, the inclusion of MRD-guided trial arms and the application of the surrogacy framework to settings where its prerequisites are not met - should be clarified before the conclusions drawn by the authors can be fully supported. At present, we believe the overall body of evidence in the literature suggests a robust MRD-PFS association, when stringent methodology is applied.
